# Objective Pain Assessment: a Key for the Management of Chronic Pain

**DOI:** 10.12688/f1000research.20441.1

**Published:** 2020-01-23

**Authors:** Xiaohan Xu, Yuguang Huang

**Affiliations:** 1 Department of Anesthesiology, Chinese Academy of Medical Sciences & Peking Union Medical College Hospital, Beijing, China

**Keywords:** chronic pain, objective assessment, functional magnetic resonance imaging, electroencephalography

## Abstract

The individual and social burdens associated with chronic pain have been escalating globally. Accurate pain measurement facilitates early diagnosis, disease progression monitoring and therapeutic efficacy evaluation, thus is a key for the management of chronic pain. Although the “golden standards” of pain measurement are self-reported scales in clinical practice, the reliability of these subjective methods could be easily affected by patients’ physiological and psychological status, as well as the assessors’ predispositions. Therefore, objective pain assessment has attracted substantial attention recently. Previous studies of functional magnetic resonance imaging (fMRI) revealed that certain cortices and subcortical areas are commonly activated in subjects suffering from pain. Dynamic pain connectome analysis also found various alterations of neural network connectivity that are correlated with the severity of clinical pain symptoms. Electroencephalograph (EEG) demonstrated suppressed spontaneous oscillations during pain experience. Spectral power and coherence analysis of EEG also identified signatures of different types of chronic pain. Furthermore, fMRI and EEG can visualize objective brain activities modulated by analgesics in a mechanism-based way, thus bridge the gaps between animal studies and clinical trials. Using fMRI and EEG, researchers are able to predict therapeutic efficacy and identify personalized optimal first-line regimens. In the future, the emergence of magnetic resonance spectroscopy and cell labelling in MRI would encourage the investigation on metabolic and cellular pain biomarkers. The incorporation of machine learning algorithms with neuroimaging or behavior analysis could further enhance the specificity and accuracy of objective pain assessments.

## Introduction

According to the recent epidemiological data, 13 to 50% of adults are experiencing chronic pain in the UK ^1^. The individual and social burdens associated with chronic pain, one of the leading causes of disability ^2^, have been escalating globally ^3^. To better understand chronic pain, we first need to address the problem of how to evaluate the severity of pain. Accurate pain measurement facilitates early diagnosis, disease progression monitoring, and therapeutic efficacy evaluation and thus is a key for the management of chronic pain. Pain is a complex multifaceted experience that has psychological, affective, and cognitive dimensions, which means that it cannot be easily characterized as a unidimensional variable. However, researchers have never stopped developing methods of evaluating pain intensity objectively. With the rapid development of neuroimaging and electrophysiological techniques, objective assessment of pain intensity has attracted substantial attention recently. The main topics of this review are the advantages, advances, and prospects of objective assessment of pain intensity.

## Limitations of subjective pain assessment

Pain is defined by the International Association for the Study of Pain as “an unpleasant sensory and emotional experience associated with actual or potential tissue damage, or described in terms of such damage” ^4^. As a subjective and complicated perception, the intensity of pain is usually evaluated in clinical settings by self-reported scales such as the Numeric Rating Scale ^5^ and Visual Analog Scale ^6^.

Such subjective methods have been considered to be “golden standards” for pain measurement ^7^; however, the accuracy and utility of self-reporting are limited under certain circumstances. First, the reliability of self-reports could be affected by a series of physiological, psychological, and environmental factors. For instance, a large number of patients with chronic pain tend to magnify their severity and hold negative attitudes ^8^. This is a phenomenon called pain catastrophizing, which has been observed in patients with migraine, rheumatic diseases, low back pain, fibromyalgia, irritable bowel syndrome, or osteoarthritis ^9^. Contrarily, underestimation may also occur, since some patients are ashamed or scared of showing their vulnerability ^7^. Second, the way to ask about pain scale could generate bias as well ^10^. In fact, assessors’ predispositions have significant impacts on the results of pain assessment ^11^. Finally, self-reporting relies on effective communications and thus is not applicable among patients under general anesthesia or with cognitive disorders. It is also difficult to get accurate verbal feedbacks from infants and young children.

Given the above limitations of self-reports, objective assessment of pain intensity has gained enormous popularity. Although absolute pain measurement is difficult because of inter-individual difference in nociceptive perception, it is still possible to identify biomarkers of relative pain intensity, which refers to the change of pain over time in an individual. Initially, physiological markers, such as blood pressure, heart rate, and pupil diameter, were used for pain evaluation ^12^. Recently, advances in neuroimaging and electrophysiological techniques have allowed more intensive and precise measurement.

## Pain signatures based on neuroimaging data

Neuroimaging techniques, such as functional magnetic resonance imaging (fMRI), are used to study functional changes in the central neural system in response to nociception. With fMRI, brain activity is indirectly quantified by the blood oxygen level–dependent (BOLD) signal, which is a surrogate indicator of regional blood oxygenation following neuronal activation ^13^.

Previous neuroimaging studies revealed that certain cortices and subcortical areas were commonly activated in subjects experiencing pain ^7^. According to a meta-analysis performed on 2,873 coordinate points, the cortices that are most likely to be activated by noxious stimuli were right anterior insula and anterior cingulate cortex, followed by left insula, bilateral secondary somatosensory cortices, prefrontal cortex, and primary somatosensory cortex/posterior parietal cortex ^14^. Additional brain regions that have been reported to be associated with pain processing included brainstem periaqueductal grey, hypothalamus, amygdala, hippocampus, and cerebellum ^15^.

Furthermore, since pain is a multidimensional experience that involves widespread brain networks, researchers tried to integrate distributed brain areas and identified potential spatiotemporal pain signatures as “dynamic pain connectome”. It is proposed that chronic pain emerges from the imbalances between functional networks ^16^. Various alterations of network connectivity were found to be correlated with the severity of clinical pain symptom ^17, 18^. Interestingly, some of the networks, such as the default mode network and salience network, are commonly involved in different types of chronic pain, indicating their pivotal roles in pain processing and central sensitizations ^19^.

However, it is still difficult to distinguish the neural response specific to noxious stimuli from that caused by other salient sensory stimuli accompanying pain. Actually, identical brain activities evoked by pain could be observed in individuals insensitive to pain ^20^. Furthermore, the relatively low temporal resolution of fMRI undermines its ability to represent fast brain activities ^21^. Therefore, the specificity of neuroimaging for pain measurement is still open to doubt, which limits its use in real-world clinical practice.

## Electrophysiological markers for pain

Scalp electroencephalography (EEG) directly reveals the spontaneous synchronized postsynaptic neuronal activity of the brain cortex with high temporal resolution ^22^. It has been widely used to detect the alterations of central neural excitation during pain processing in recent years.

Resting EEG demonstrated suppressed spontaneous oscillations in healthy volunteers experiencing pain ^23, 24^. Furthermore, the peak alpha frequency recorded at bilateral temporal scalp was found to be correlated with subjectively rated pain scores and thus potentially reflects the pain intensity ^24– 26^. In patients with chronic pain following spinal cord injury ^27, 28^ and chronic pancreatitis ^29, 30^, a lowered dominant peak frequency was commonly observed. This result was consistent with the phenomenon of thalamocortical dysrhythmia (TCD), which has been discovered in a series of neurological or psychiatric conditions ^31^.

EEG signals from different electrodes reflect the network of synchronized neurons, which provides the basis for the functional connectivity analysis by resting EEG ^32^. Using the spectral power and coherence analysis, researchers are also able to explore the dynamics of the brain networks involved in pain processing. For instance, patients with fibromyalgia exhibited greater beta power in the right middle frontal lobe and midcingulate gyrus, augmented theta power in prefrontal and anterior cingulate cortices, and increased centro-parietal coherence at the left hemisphere on theta- and beta-bands ^33– 35^.

Additionally, since resting EEG may be confounded by a series of brain processing other than pain, EEG response to evoked potentials provides a more specific insight into nociceptive sources of localization ^36^. As the channels of electrode cap increase to more than 120, the spatial resolution can be improved accordingly ^37^. With multichannel matching pursuit, brain source connectivity can also be investigated ^38– 40^. Similarly, researchers also tried to evaluate the spinal nociceptive reflex by electromyographic (EMG) response, which provides clues for mechanisms of nociception transmission and central sensitization ^41^.

Compared with neuroimaging, the electrophysiological method has a unique advantage. Since no large equipment is required, it can be conveniently performed in wards or operating rooms. Therefore, physicians can obtain a continuous record of primary cortical activities in real clinical scenarios using EEG.

## The use of functional magnetic resonance imaging and electroencephalography in drug development and personalized treatment

Besides their important roles in pain assessments, fMRI and EEG have significant advantages in analgesic development and personalized treatment. First, fMRI and EEG can visualize objective brain activities modulated by analgesics in a mechanism-based way ^42^. The application of quantitative analysis in fMRI and EEG further yields reliable information on dose-efficacy relationships ^43^. Importantly, the valuable neuroimaging evidence bridges the gaps between animal studies and clinical trials. It was recommended to perform neuroimaging studies on a small sample size of volunteers in the early stage of drug development since it would provide clues for whether to further conduct costly and time-consuming trials ^44^. Up to now, fMRI and EEG have been successfully used in several preliminary double-blinded, randomized clinical trials for analgesics ^44, 45^.

Second, it is possible to predict therapeutic efficacy and identify optimal first-line regimens by using fMRI and EEG. Conventionally, physicians tended to choose therapeutic strategies based on the severity and etiology of pain. Nowadays, fMRI and EEG provide opportunities for personized mechanism-targeted treatment. For instance, an fMRI study showed that baseline reward circuitry activity was predictive for opioid analgesic responses, which enables the stratification of patients before treatment ^46^. Similarly, another fMRI study found that right midfrontal gyrus connectivity could predict placebo responses, which allows physicians to distinguish drug efficacy from placebo analgesia ^47^.

## Further directions

Despite the exciting progress made in pain assessment by neuroimaging and electrophysical techniques, this is not the whole picture. Actually, a wide range of biochemical, molecular, and cellular pain biomarkers await to be explored ^7, 48– 50^. A variety of advanced techniques have been used extensively in pain research to find such biomarkers. The emergence of magnetic resonance spectroscopy allows researchers to obtain quantitative information about brain metabolites ^51^. By labelling immune cells with superparamagnetic particles of iron oxide (SPIO) in MRI, the trafficking of microglia and astrocytes can be well demonstrated, which allows *in vivo* imaging of human neuroinflammation ^51^. Positron emission tomography and single-photon emission computed tomography can help visualize the distribution and activity of nociceptive receptors by radiolabeling their ligands ^21^.

Another promising research direction is machine learning. Given the huge amount of data contained in neuroimaging, the incorporation of machine-learning algorithms could facilitate image recognition and data analysis. Machine-learning algorithms have been successfully used to distinguish human brain responses to painful stimuli ^52^. Additionally, machine learning demonstrates great prospects in pain-related behavior analysis, such as automatic assessments of pain on the basis of facial expressions.

In conclusion, objective pain assessment is a key for chronic pain management. Greater efforts should be made to identify and validate nociceptive biomarkers. In the future, the integration of a wide range of biomarkers would provide a comprehensive understanding of pain processing and treatment.

## Abbreviations

EEG, electroencephalography; fMRI, functional magnetic resonance imaging
